# N4-acetylcytidine modification of LINC02802 promotes non-small cell lung cancer progression by modulating mitochondrial NAD+/NADH ratio

**DOI:** 10.7150/ijbs.116639

**Published:** 2025-07-28

**Authors:** Yixiao Yuan, Dahang Zhang, Juan Wang, Lin Tang, Yaowu Duan, Lincan Duan, Xiulin Jiang

**Affiliations:** 1 Department of Thoracic Surgery, Yunnan Cancer Hospital, The Third Affiliated Hospital of Kunming Medical University, Peking University Cancer Hospital Yunnan, Kunming, Yunan, 650118, China.; 2 Department of Systems Biology, Beckman Research Institute of City of Hope, Monrovia, CA 91016, USA.; 3 Department of Thoracic Surgery, Pu'er People's Hospital, Pu'er, Yunan, China.

**Keywords:** LINC02802, miR-1976, SLC25A51, NAD+/NADH ratio, mitochondrial metabolism, NSCLC

## Abstract

Long non-coding RNAs (lncRNAs) have emerged as key regulators of cancer progression through their interaction with microRNAs and modulation of gene expression. However, their role in mitochondrial metabolism, particularly in non-small cell lung cancer (NSCLC), remains poorly defined. In this study, we found that LINC02802 was significantly upregulated in NSCLC tissues and associated with poor prognosis. Mechanistically, LINC02802 acts as a competing endogenous RNA (ceRNA) for miR-1976, thereby relieving the suppression of solute carrier family 25 member 51(SLC25A51). Elevated SLC25A51 enhances mitochondrial NAD^+^ import, leading to an increased NAD^+^/NADH ratio and promoting oxidative TCA cycle flux. Functionally, this shift supports tumor cell proliferation and migration. Rescue experiments confirmed that the oncogenic effect of LINC02802 is dependent on the miR-1976/SLC25A51 axis. Interestingly, either silencing LINC02802 with antisense oligonucleotides (ASOs) or treating cells with fludarabine phosphate, an SLC25A51 inhibitor, successfully reversed cisplatin resistance in lung cancer cells. Our findings reveal a novel lncRNA-microRNA-metabolic axis wherein LINC02802 facilitates NSCLC progression by reprogramming mitochondrial metabolism via miR-1976-mediated upregulation of SLC25A51. Targeting this axis may offer therapeutic potential for metabolic intervention in NSCLC.

## Introduction

Non-small cell lung cancer (NSCLC) accounts for approximately 85% of all lung cancer cases and remains one of the leading causes of cancer-related mortality worldwide 1-3. Despite significant advances in therapeutic strategies—including surgery, chemotherapy, targeted therapy, and immunotherapy—the prognosis for NSCLC patients remains poor, largely due to late-stage diagnosis and the frequent development of drug resistance 3-5. These clinical challenges highlight the urgent need for more effective early diagnostic markers and therapeutic.

In recent years, substantial efforts have been made to identify molecular biomarkers associated with NSCLC, and several candidates have shown potential for diagnosis, prognosis, or treatment response prediction 6. However, most of these markers are not directly linked to mitochondrial metabolism, which plays a crucial role in tumor growth and survival. Mitochondrial function, particularly the regulation of redox balance and energy production through the NAD⁺/NADH ratio, is essential for sustaining cancer cell proliferation and metabolic plasticity 7.

Non-coding RNAs (ncRNAs) play a critical regulatory role in the development and progression of lung cancer 8. Increasing evidence has demonstrated that various types of ncRNAs, including long non-coding RNAs (lncRNAs), circular RNAs (circRNAs), and microRNAs (miRNAs), are deeply involved in modulating oncogenes and tumor suppressor genes, influencing key biological processes such as cell proliferation, apoptosis, migration, invasion, and metabolism 9, 10. Yet, the contribution of long non-coding RNAs (lncRNAs) to the modulation of mitochondrial metabolism in NSCLC remains largely unexplored. Given the limited efficacy of current therapies, it is imperative to identify novel lncRNA biomarkers that regulate mitochondrial metabolism, as these may not only overcome the shortcomings of existing treatments but also fulfill the unmet need for early detection of lung cancer. In particular, targeting lncRNAs involved in the mitochondrial NAD⁺/NADH balance may offer a promising avenue for developing metabolic therapies in NSCLC.

LINC02802 is a long non-coding RNA (lncRNA) that has recently emerged as a potential regulator in cancer biology. Although its functions remain incompletely characterized, accumulating evidence suggests that LINC02802 is involved in modulating cellular metabolism and tumor progression 11. SLC25A51 is a recently identified member of the solute carrier family 25 (SLC25), which encodes mitochondrial carrier proteins responsible for transporting various metabolites across the inner mitochondrial membrane 12. Notably, SLC25A51 has been recognized as the major mitochondrial NAD⁺ transporter in mammalian cells, playing a pivotal role in maintaining the mitochondrial NAD⁺ pool 13, which is essential for numerous metabolic processes, including oxidative phosphorylation, the tricarboxylic acid (TCA) cycle, and mitochondrial protein deacetylation 14. Dysregulation of NAD⁺ metabolism has been increasingly implicated in cancer development and progression, suggesting that SLC25A51 may serve as a critical mediator linking mitochondrial metabolism to tumorigenesis 15. However, the precise role of SLC25A51 in non-small cell lung cancer (NSCLC) remains largely unexplored.

In summary, while accumulating evidence highlights the pivotal role of lncRNAs in tumor metabolism and mitochondrial function, the impact of epigenetic modifications such as N4-acetylcytidine (ac4C) on lncRNA-mediated metabolic reprogramming in cancer remains largely unexplored. In this study, we identify LINC02802 as a novel ac4C-modified lncRNA that contributes to non-small cell lung cancer progression. We further elucidate a mechanistic axis in which LINC02802 modulates the mitochondrial NAD⁺/NADH ratio by acting as a ceRNA for miR-1976, thereby upregulating the mitochondrial transporter SLC25A51. These findings uncover a previously unrecognized layer of epitranscriptomic regulation in NSCLC and provide new insights into the interplay between RNA modifications and cancer metabolism.

## Materials and methods

### Expression and prognosis analysis of LINC02802 in cancer

We initially downloaded transcriptomic data from The Cancer Genome Atlas (TCGA) and the Genotype-Tissue Expression (GTEx) database 16. To further validate the expression of LINC02802 in different cancer types, we employed the "DESeq2" or "edgeR" packages in R for differential expression analysis 17, 18.

### Tissue microarrays

Lung cancer tissue microarrays and corresponding normal control tissue microarrays were purchased from Servicebio, Wuhan. These arrays included various stages of lung cancer tissues as well as normal lung tissues, aimed at analyzing the expression differences of LINC02802 between tumor and normal tissues. *In situ* hybridization (ISH) technology was employed to detect the expression levels of LINC02802 in lung cancer tissues and normal control tissues. Expression levels were observed under a microscope, and the staining intensity in the nuclei or cytoplasm was assessed, with semi-quantitative analysis conducted using ImageJ or other quantitative software. The expression levels of LINC02802 in normal control tissues were compared to those in lung cancer tissues, and non-parametric tests were applied to analyze the significance of expression differences, with a p-value of less than 0.05 considered statistically significant.

### Clinical samples

Clinical samples were obtained from the Third Affiliated Hospital of Kunming Medical University, comprising 15 paraffin-embedded lung cancer samples that were histopathologically and clinically diagnosed at Third Affiliated Hospital of Kunming Medical University from 2018 to 2022. After surgery, the patients received regular follow-up. This study was approved by the institutional research ethics committee of Third Affiliated Hospital of Kunming Medical University.

### Lentivirus package

A three-plasmid packaging system was employed, including the packaging plasmids pMD2.G and pSPAX, and either the target plasmid or an empty vector such as pLKO.1 or pCDH-CMV-Puro. Lentiviral packaging was carried out using the calcium chloride transfection method, following the standard PEI transfection protocol, and transfected into HEK-293T cells. Cell culture supernatants were collected at 24 and 48 hours post-medium change. The collected supernatants were filtered through 0.45 μm sterile filters and aliquoted as needed. For long-term storage, the virus stocks were stored at -80°C, while for short-term use, they could be kept at 4°C for up to one week.

### QRT-PCR assay

RNA was extracted from human bronchial epithelial cells (BEAS-2B cells) and NSCLC cancer cells, reverse transcribed into cDNA, and then their expression levels were identified using qPCR. In this study, the reference gene was β-actin, The primer information used in this study is provided in Supplementary Table 1.

### Cell culture and migration assay

This study included the normal lung epithelial cell line BEAS-2B (human bronchial epithelial cells) as well as NSCLC cell lines from ATCC. The BEAS-2B cells were cultured in BEGM supplemented with 10% fetal bovine serum (FBS), while NSCLC cell lines were cultured in RPMI-1640 or DMEM medium with 10% FBS and 1% penicillin-streptomycin (P/S) to prevent bacterial contamination. The cell lines were maintained in a 37°C incubator with 5% CO₂, with medium changes every 2-3 days to ensure an appropriate growth environment for the cells.

### Western blot assay

Cells were collected by centrifugation, and the pellet was resuspended and lysed thoroughly with an appropriate volume of 2× SDS loading buffer. The lysates were then boiled at 98°C for 15 minutes in a dry bath. Equal amounts of protein samples were loaded onto pre-cast SDS-PAGE gels. In addition, 4 μL of protein marker was loaded into the lanes adjacent to the samples. Electrophoresis was performed at a constant voltage of 80 V for 30 minutes, followed by 120 V for 1 hour. Proteins were transferred onto NC membranes at a constant voltage of 100 V for 1 hour. After primary antibody incubation, the membranes were washed three times with TBST (TBS containing 0.1% Tween-20), each. The antibody information used in this study is provided in Supplementary Table 2.

### Cell proliferation assay

NSCLC cells that survived 48 hours of puromycin selection following lentiviral infection, as well as control (uninfected) cells, were counted. The cells were then diluted to a concentration of 10,000 cells/mL, and 10,000 cells were seeded into each well of a 24-well plate. Each treatment group included three replicate wells. The day of seeding was designated as Day 0. Cell numbers were counted at the same time points on Days 2, 4, and 6 to assess the effect of LINC02802 knockdown on cell proliferation.

### Colony formation assay

Following puromycin selection, NSCLC cells that survived were counted and seeded into 6-well plates at a density of 600 cells per well in 2 mL of complete medium, with three replicate wells per group. After one week of incubation, colony formation was examined under a microscope, and the number of colonies in each group was quantified.

### Seahorse energy metabolism analysis

Seahorse energy metabolism analysis was performed based on protocols established in previously published studies 14. To evaluate glycolytic activity and oxidative phosphorylation levels, the Agilent Seahorse XF Cell Mito Stress Test Kit was used. Cells were seeded into Seahorse XF96 cell culture microplates at a density of 1 x 10⁶ cells/mL in advance. Taking the measurement of extracellular acidification rate (ECAR) as an example, cells were pretreated according to experimental requirements and incubated in Seahorse XF glycolysis stress test medium containing glucose, glutamine, sodium pyruvate, and HEPES buffer. Basal ECAR levels were recorded over three measurement cycles. Glucose, oligomycin (Oligo), and 2-deoxy-D-glucose (2-DG) were subsequently added to monitor real-time changes in glycolytic activity. For oxygen consumption rate (OCR) measurement, a series of compounds including oligomycin and FCCP were sequentially added to assess real-time changes in OCR, reflecting mitochondrial respiration activity. The commercially available assay kit information used in this study is provided in Supplementary Table 3.

### Dual-luciferase reporter assay

H1299 cells or mouse macrophages were co-transfected with a luciferase reporter plasmid containing the target gene, the PRL-TK-Renilla plasmid as an internal control, and appropriate amounts of activator expression vectors, along with LINC02802 WT constructs or mutants. The total amount of DNA was equalized across all transfection conditions using an empty vector. Approximately 24 hours post-transfection, cells were lysed using a passive lysis buffer. Luciferase activity in the cell lysates was measured using the Dual-Luciferase® Reporter Assay System (Promega). Firefly luciferase activity representing the target gene was normalized to Renilla luciferase activity for each sample.

### Detection of TCA cycle intermediates

A commercially available assay kit was used to detect fumarate levels following the manufacturer's instructions. Collect the cell suspension and centrifuge at 200 × g for 5 minutes. Discard the supernatant and lyse the pellet in 100 μL of cell lysis buffer on ice for 30 minutes. Centrifuge the lysate at 12,000 × g for 10 minutes at 4°C and collect the supernatant. Prepare a 96-well plate by adding 100 μL of Fumarate Developer to each well. Quickly add 100 μL of sample supernatant to the corresponding wells. Measure absorbance at 450 nm using a microplate reader. The commercially available assay kit information used in this study is provided in Supplementary Table 3.

### Xenograft tumor formation assay

Male nude mice aged 4-6 weeks were subcutaneously injected with H1299 cell lines (1×10^6^ cells), when the xenograft tumors reached to 50 mm^3^ of volume, they were randomly divided into two groups. The mice were injected with miR Ctrl or mimics (5 nM) around the tumor twice per week. Nude mice were monitored every other day, xenograft tumor weights and volumes were measured with a sliding caliper, and tumor volumes were calculated using the formula (L×W^2^)/2. All animals were kept in a SPF environment and the protocols were pre-approved and conducted under the policy of Animal care and Use Committee at the The Third Affiliated Hospital of Kunming Medical University, Peking University Cancer Hospital Yunnan.

### Statistical analysis

Student's *t*-test was used to evaluate biological significance in experimental comparisons. The log-rank test was applied to assess differences in survival curves and their statistical relevance. In this study, “n.s.” indicates a lack of biological statistical significance (not significant), whereas *P* < 0.05 was considered statistically significant. The levels of significance were denoted as follows: *P* < 0.05 (*), P < 0.01 (**)**,** and *P* ***<*** 0.001 (***).

## Results

### LINC02802 was highly expressed in NSCLC

**Figure 1:**
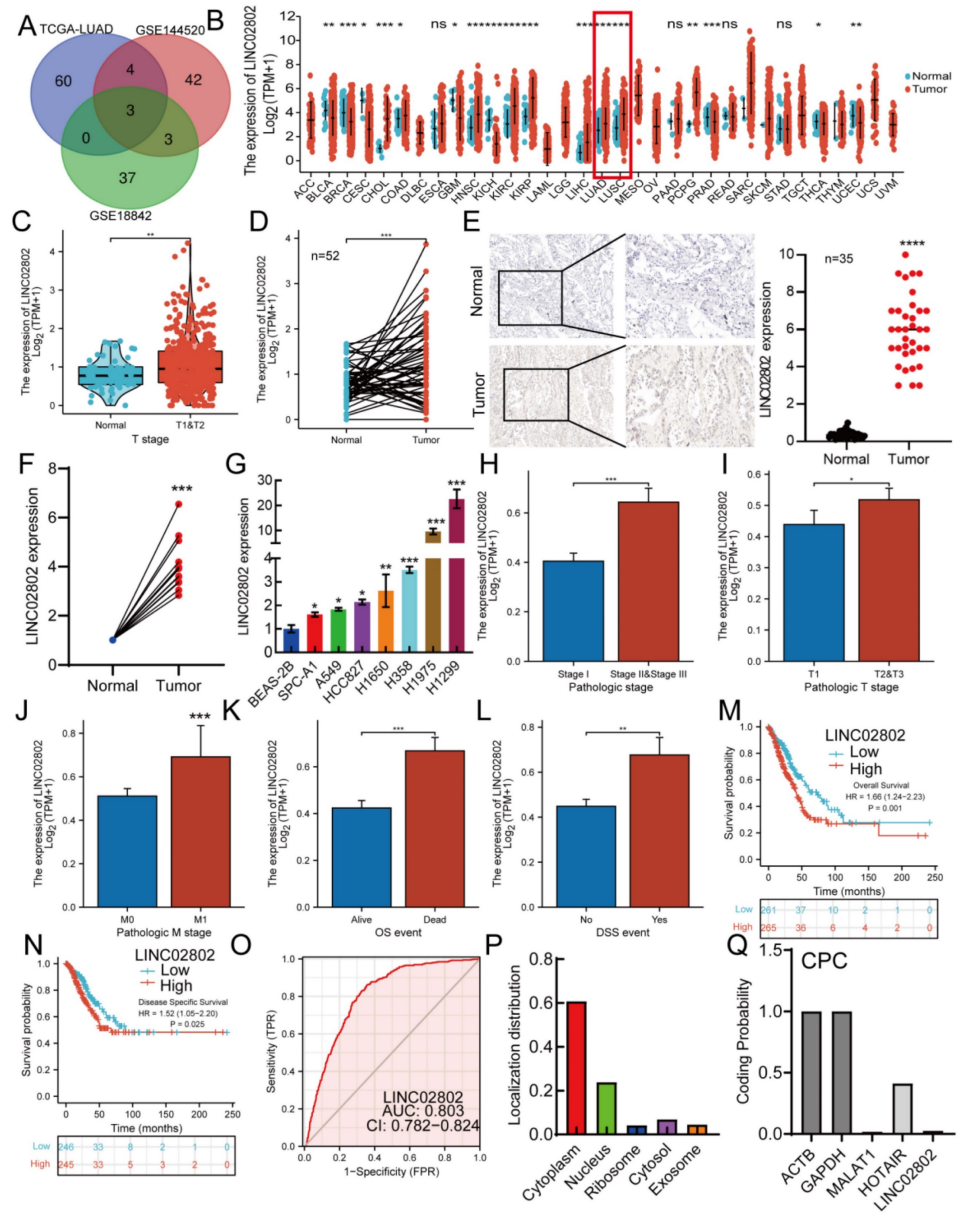
** LINC02802 is highly expressed in lung cancer.** (A) Integration of TCGA and GEO datasets to identify LINC02802 as a potential lung cancer-associated lncRNA. (B) Combined TCGA and GTEx analysis of LINC02802 expression across multiple cancer types. (C-D) Analysis of LINC02802 expression in lung adenocarcinoma (LUAD) based on TCGA data. (n=52). (E-F) Validation of LINC02802 expression in lung cancer tissues using *in situ* hybridization (ISH) and qPCR, ((E) n=35, (F) n=10). (G) qPCR analysis of LINC02802 expression in lung cancer cell lines compared to normal lung epithelial cells. (H-L) Clinical relevance of LINC02802 expression in LUAD patients analyzed using the TCGA database. (M-N) Prognostic significance of LINC02802 expression in LUAD assessed by Kaplan-Meier survival analysis using TCGA data. (O) ROC curve analysis evaluating the diagnostic performance of LINC02802 in LUAD. (P-Q) Analysis of LINC02802 subcellular localization and coding potential using CPC and related databases. *P < 0.05, **P < 0.01, ***P < 0.001.

We integrated TCGA and GEO datasets: (1) TCGA-LUAD: identified lncRNAs significantly upregulated in lung adenocarcinoma (LogFC > 4, p < 0.01); (2) GSE144520: Compared with sensitive A549 lung cancer cells, lncRNAs upregulated in cisplatin-resistant A549 cells (LogFC > 4, p < 0.01); (3) GSE18842: Transcriptome sequencing dataset of lung cancer tissues and normal tissues, screening and identifying lncRNAs upregulated in cisplatin-resistant lung cancer cells, intersecting to obtain three long non-coding RNAs: LINC02802, NKILA, and SENCR** (Figure 1A)**. Among them, LINC02802 was the most significantly upregulated and had not been reported in the progression of NSCLC, so we identified it as a potential target gene for further study of its function in lung adenocarcinoma. We analyzed the expression of LINC02802 in pan-cancer using transcriptome data from TCGA and GTEx, showing that LINC02802 expression was higher in CHOL, ESCA, HNSC, KIRC, KIRP, LIHC, LUAD, LUSC, and PCPG than in control groups** (Figure 1B)**. We analyzed the paired expression in cancer tissues and control tissues from the TCGA database, confirming that LINC02802 expression was higher in HNSC, KIRC, KIRP, LIHC, LUAD, and LUSC than in control groups** (Figure S1A)**. Furthermore, prognosis analysis showed that LINC02802 expression correlated with the prognosis of many cancers. Specifically, in lung adenocarcinoma, we found that LINC02802 was significantly upregulated** (Figure 1C-1D)**. To validate the database analysis results, we used *in situ* hybridization to detect expression of LINC02802 in lung cancer tissue microarrays ( comprised of 35 lung paracancer tissues and 35 lung cancer tissues annotated with clinical and pathological information (Wuhan Servicebio, China) , results showing that LINC02802 expression was significantly higher in NSCLC tissues than in adjacent tissues and mainly located in the cytoplasm** (Figure 1E).**(P < 0.001), Additionally, we collected and extracted RNA from 15 pairs of fresh NSCLC and adjacent tissue samples from Yunnan Cancer Hospital (Informed consent was obtained from patients for all sample collections) and using qRT-PCR experiments to validation the expression of LINC02802 in lung cancer, results showed that LINC02802 expression was higher in NSCLC tissues than in adjacent tissues, consistent with *in situ* hybridization and bioinformatics analysis results **(Figure 1F)** (P < 0.01). Further analysis of LINC02802 expression in lung cancer cell lines showed that, compared with normal lung epithelial cells, LINC02802 was significantly upregulated in most lung cancer cell lines, especially H1975 and H1299** (Figure 1G)**.

Correlation analysis between LINC02802 expression and clinical relevance in lung cancer patients showed that LINC02802 expression was closely related to pathological stage, TM stage, OS, and DSS event time in lung adenocarcinoma patients** (Figure 1H-1L)**. Prognosis analysis indicated that high LINC02802 expression suggested poorer survival in pan-cancer patients** (Figure 1M-1N)**. ROC curve analysis showed an AUC value of 0.803 for LINC02802, indicating its diagnostic value in lung adenocarcinoma** (Figure 1O)**. LINC02802 also exhibits strong predictive value in HCC, BRCA, and HNSC **(Figure S2A-S2C)**. Database analysis further revealed that LINC02802 was primarily located in the cytoplasm, consistent with *in situ* hybridization results, and protein-coding potential analysis showed that LINC02802 did not have protein-coding potential** (Figure 1P-1Q)**. Pan-cancer prognosis analysis showed that patients with high LINC02802 expression had significantly shorter overall survival and disease-free survival than those with low expression** (Figure S1B-1E)**. Considering that LINC02802 influences the prognosis of lung cancer patients, we further investigated whether it also affects the tumor immune microenvironment and thereby influences patient outcomes. Analysis of TCGA lung cancer data revealed that LINC02802 expression was not significantly correlated with either the immune score or stromal score **(Figure S2D)**, suggesting that alterations in the lung cancer immune microenvironment may not contribute to abnormal LINC02802 expression. This implies that other molecular mechanisms are likely involved in regulating LINC02802 expression. In summary, these studies indicate that LINC02802 is upregulated in lung adenocarcinoma and significantly associated with patient prognosis, suggesting that LINC02802 may be involved in the progression of lung adenocarcinoma.

### Knockdown of LINC02802 inhibits the progression of lung cancer both *in vitro* and *in vivo*

**Figure 2:**
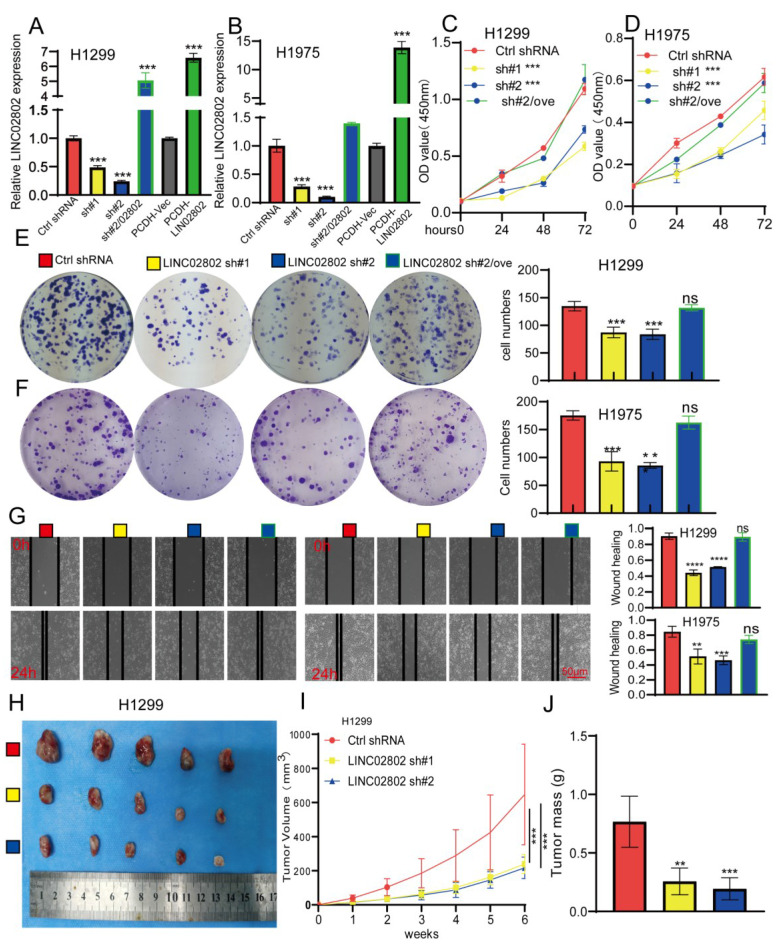
** Knockdown of LINC02802 inhibits lung cancer cell proliferation and migration.** (A-B) qPCR analysis confirming LINC02802 knockdown efficiency in lung cancer cell lines. (C-D) CCK-8 assays assessing the effect of LINC02802 knockdown on cell proliferation. (E-F) Colony formation assays evaluating the impact of LINC02802 knockdown on clonogenic potential. (G) Wound healing assay assessing the effects of LINC02802 knockdown on migration and invasion. (H-J) LINC02802 knockdown reduces tumor formation in xenograft mouse models. Representative images of tumors (H), tumor volumes (I), and tumor weights (J) are shown. (n = 5 mice per group). Data are presented as mean ± SD (bar plots). *P < 0.05; **P < 0.01; ***P < 0.001; ns not significant.

To further study the biological function of LINC02802 in the progression of lung adenocarcinoma, we selected H1975 and H1299 cell lines with the highest LINC02802 expression for further functional experiments. We constructed stable LINC02802 knockdown cell lines using shRNA, and qRT-PCR experiments verified the knockdown efficiency, showing that both shRNAs significantly inhibited LINC02802 expression in lung cancer cell lines** (Figure 2A-2B)**. CCK8 experiments confirmed that LINC02802 knockdown inhibited lung cancer cell proliferation **(Figure 2C-2F)**, and colony formation experiments showed that LINC02802 knockdown significantly inhibited cell colony formation abilities **(Figure 2E-2F)**. Wound healing and transwell assays indicated that LINC02802 knockdown significantly inhibited cell migration and invasion abilities of lung cancer cells** (Figure 2G and Figure S2E)**. Importantly, LINC02802 overexpression partially rescued the inhibitory effects caused by LINC02802 knockdown on cell proliferation and migration, indicating that LINC02802 plays a crucial role in lung cancer cell proliferation and metastasis. Cisplatin (DDP) is a commonly used chemotherapy drug for lung cancer; however, resistance to cisplatin is a major reason for poor therapeutic outcomes 19. Numerous studies have shown that aberrant epigenetic modifications, including abnormal lncRNA expression, play crucial roles in regulating cisplatin resistance in lung cancer 20. Given that LINC02802 was found to be highly expressed in cisplatin-resistant cell lines, it is hypothesized that the elevated expression of LINC02802 may be associated with cisplatin resistance in lung cancer. To further investigate whether LINC02802 affects cisplatin resistance, we conducted flow cytometry assays, which revealed that knockdown of LINC02802 significantly induced apoptosis in lung cancer cells **(Figure S3A-S3B)**. More importantly, knockdown of LINC02802 increased the sensitivity of lung cancer cells to cisplatin, leading to higher cell death rates **(Figure S3C-S3D)**. To further investigate the biological function of LINC02802 *in vivo*, a xenograft model using H1299 cells was established. Compared with the control group, knockdown of LINC02802 significantly suppressed tumor growth, as evidenced by reduced tumor size and volume **(Figure 2H-2J)**. These results collectively indicate that LINC02802 is highly expressed in lung cancer and plays a promotive role in lung cancer progression.

### LINC02802 exerts its function by sponging miRNA-1976 in lung cancer cells

**Figure 3:**
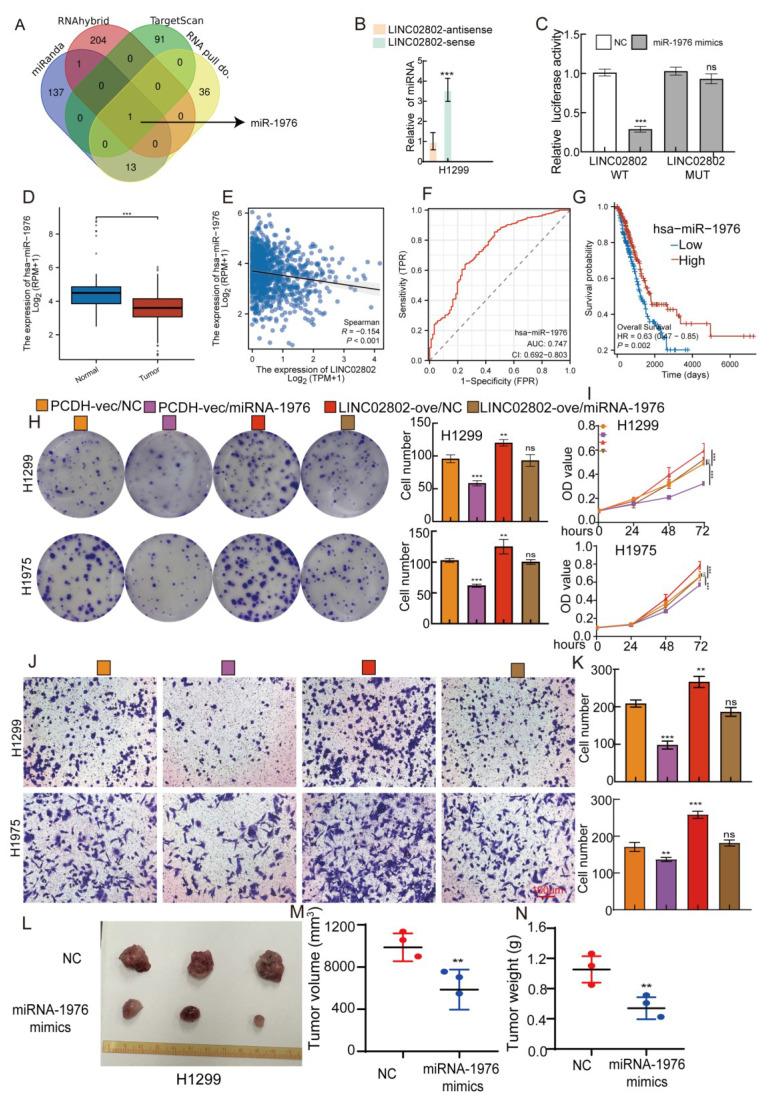
** The LINC02802/miR-1976 axis regulates proliferation and migration in lung adenocarcinoma.** (A) Bioinformatics analysis combined with RNA pull-down assays to identify miRNAs interacting with LINC02802. (B) RNA pull-down using a LINC02802-specific probe to validate its binding with miR-1976. (C) Dual-luciferase reporter assays confirming direct interaction between LINC02802 and miR-1976. (D-E) TCGA-based analysis of miR-1976 expression and its correlation with LINC02802 in LUAD. (F) ROC curve evaluating the diagnostic potential of miR-1976 in LUAD. (G) Kaplan-Meier survival analysis of miR-1976 in LUAD. (H-I) CCK-8 and colony formation assays demonstrating the regulatory effect of the LINC02802/miR-1976 axis on cell proliferation. (J-K) Transwell assays evaluating the impact of the LINC02802/miR-1976 axis on cell migration and invasion. (L-N) miR-1976 mimics suppress tumor formation *in vivo* by H1299 cells, Representative images (L), tumor volume (M), and tumor weight (N) are shown. (n = 3 mice per group) Data are presented as mean ± SD (bar plots). sh#1=LINC02802 shRNA#1, sh#2=LINC02802 shRNA#2, Ove=LINC02802 Overexpression. NC= negative control, *P < 0.05; **P < 0.01; ***P < 0.001; ns not significant.

As previously mentioned, LINC02802 is primarily localized in the cytoplasm, suggesting that it may function as a miRNA sponge to regulate gene expression. Based on this, we used various bioinformatics databases (miRanda, RNAhybrid and Targetscan) to predict miRNAs that could interact with LINC02802. These predicted results were then intersected with the miRNAs pulled down by LINC02802 in the RNA pull-down assay. The intersection of these predictions revealed that miRNA-1976 could interact with LINC02802**(Figure 3A)**. Further RNA pull down experiments showed that LINC02802 significantly pull down miRNA-1976 **(Figure 3B)**. Dual-luciferase reporter assays confirmed that miRNA-1976 could significantly inhibit the activity of LINC02802, indicating an interaction between miRNA-1976 and LINC02802. Mutations in the binding site of LINC02802 restored the inhibitory effect of miRNA-1976 mimics on luciferase activity **(Figure 3C)**. To further explore the clinical significance and expression levels of miRNA-1976 in lung cancer tissues, we analyzed the TCGA database and found that miRNA-1976 was downregulated in lung adenocarcinoma **(Figure 3D)**, with its expression negatively correlated with LINC02802 **(Figure 3E)**. ROC curve analysis showed an AUC value of 0.747 **(Figure 3F)**, and patients with higher miRNA-1976 expression had better overall survival rates **(Figure 3G and Figure S4A-4B)**. To further validate the role of the LINC02802/miRNA-1976 interaction in lung cancer progression, we conducted rescue experiments *in vitro*. Clonogenic and CCK8 assays showed that overexpression of miRNA-1976 inhibited lung cancer cell proliferation and growth, while overexpression of LINC02802 rescued the inhibitory effect caused by overexpression of miRNA-1976 **(Figure 3H-3I)**. Transwell assays also showed that overexpression of miRNA-1976 inhibited lung cancer cell migration, and overexpression of LINC02802 rescued this effect **(Figure 3J-3K)**. Furthermore, our xenograft experiments in nude mice demonstrated that overexpression of miRNA-1976 significantly inhibited tumor growth, reducing both tumor volume and weight **(Figure 3L-3N)**. These data suggest that LINC02802 acts as a molecular sponge for miRNA-1976, inhibiting its function and promoting the malignant phenotype of lung adenocarcinoma cells.

### LINC02802 enhances SLC25A51 expression by sponging miRNA-1976 in lung cancer cells

**Figure 4:**
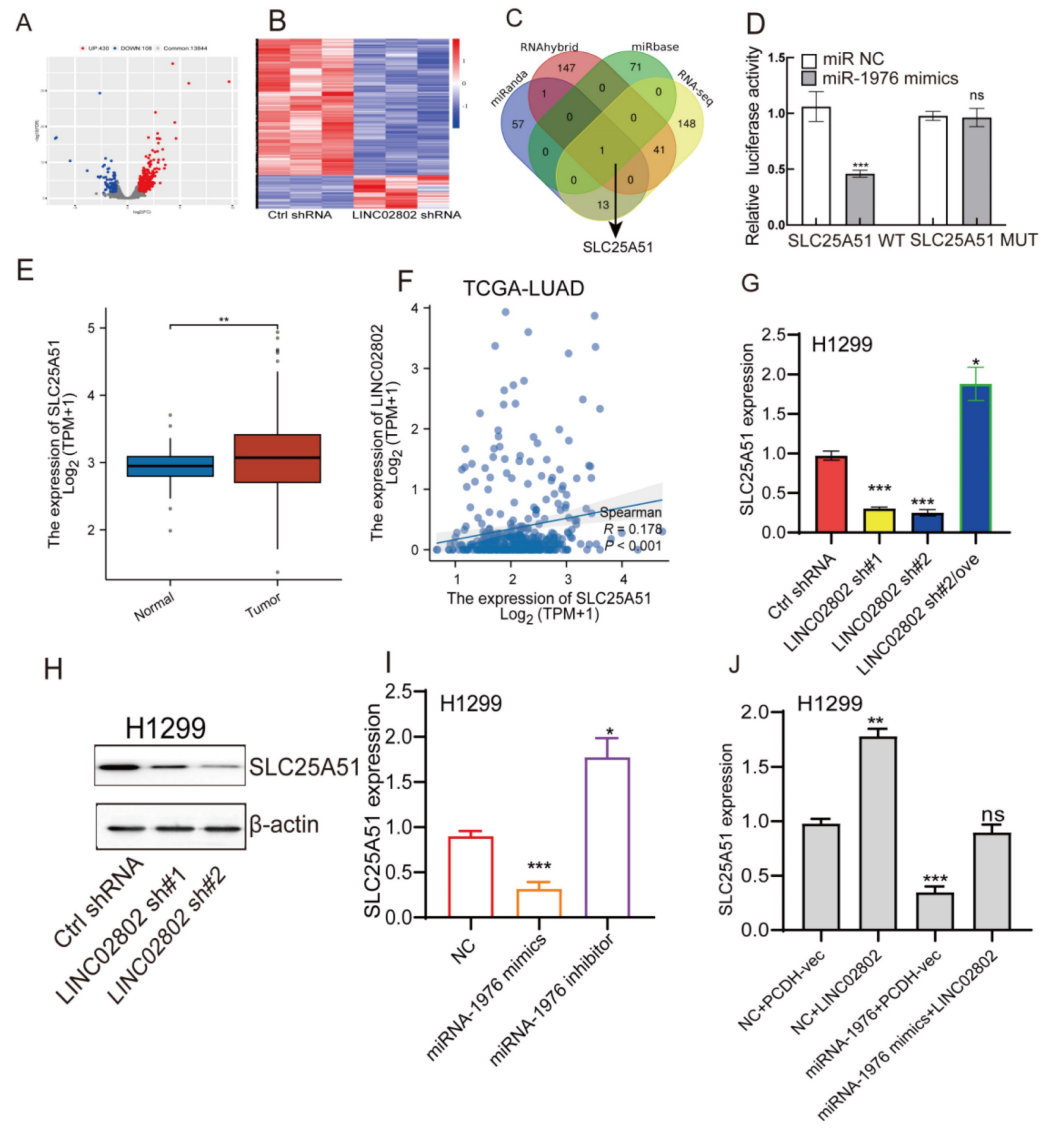
** SLC25A51 is a downstream target of the LINC02802/miR-1976 axis.** (A-B) Volcano plot and heatmap showing differentially expressed genes identified by transcriptome sequencing after LINC02802 knockdown. (C) RNA-seq and bioinformatic analysis to predict miR-1976 downstream target genes. (D) Dual-luciferase reporter assays confirming the binding of miR-1976 to the 3′UTR of SLC25A51. (E) TCGA-based expression analysis of SLC25A51 in LUAD. (F) Correlation analysis between LINC02802 and SLC25A51 expression in LUAD. (G-I) Expression changes of SLC25A51 following LINC02802 knockdown or miR-1976 overexpression. (J) Expression analysis of SLC25A51 after overexpression of LINC02802 and miR-1976. Data are presented as mean ± SD (bar plots). *P < 0.05; **P < 0.01; ***P < 0.001; ns not significant.

To identify the downstream target genes regulated by LINC02802, we performed transcriptome sequencing on LINC02802 knockdown cell lines. Differential expression analysis revealed that 538 genes were significantly altered (log2FC > 1.5 or log2FC < -1.5 p < 0.05)** (Figure 4A-4B)**. GSEA enrichment analyses of the downregulated genes indicated that they were predominantly enriched in the oxidative phosphorylation and fatty acid metabolism signaling pathway** (Figure S4C)**, suggesting a potential role of LINC02802 in the regulation of mitochondrial metabolism. To further clarify the downstream targets of the LINC02802/miR-1976 signaling axis, we used three different bioinformatics databases to predict target genes of miR-1976. These predicted targets were then intersected with differentially expressed genes from the LINC02802 knockdown RNA-seq data (log2FC > 1.5 or log2FC < -1.5, p < 0.05), resulting in the identification of SLC25A51 as a potential direct target** (Figure 4C)**. Dual-luciferase reporter assays confirmed the interaction between miRNA-1976 and SLC25A51 **(Figure 4D)**. Compared to normal lung tissues, SLC25A51 was significantly upregulated in lung adenocarcinoma tissues, with its expression positively correlated with LINC02802 **(Figure 4E-4F)**.

To further validate whether SLC25A51 acts as a downstream target gene of the LINC02802/miR-1976 axis, we employed qRT-PCR and Western blot experiments. The results revealed that knockdown of LINC02802 significantly downregulated the expression levels of SLC25A51, both at the RNA and protein levels. Overexpression of LINC02802 was able to rescue the downregulation of SLC25A51 caused by LINC02802 knockdown** (Figure 4G-4H)**. Additionally, we found that overexpression of miRNA-1976 significantly downregulated SLC25A51 expression, while inhibition of endogenous miRNA-1976 markedly upregulated SLC25A51 expression **(Figure 4I)**. Moreover, overexpression of LINC02802 can rescue the inhibitory effect of miRNA-1976 on SLC25A51 expression **(Figure 4J)**. In conclusion, these results demonstrate that LINC02802 functions as a molecular sponge for miRNA-1976, thereby relieving the inhibitory effects of miRNA-1976 on the expression of the downstream target gene SLC25A51 and consequently upregulating SLC25A51 expression levels.

### Knockdown of SLC25A51 suppresses NSCLC progression

**Figure 5:**
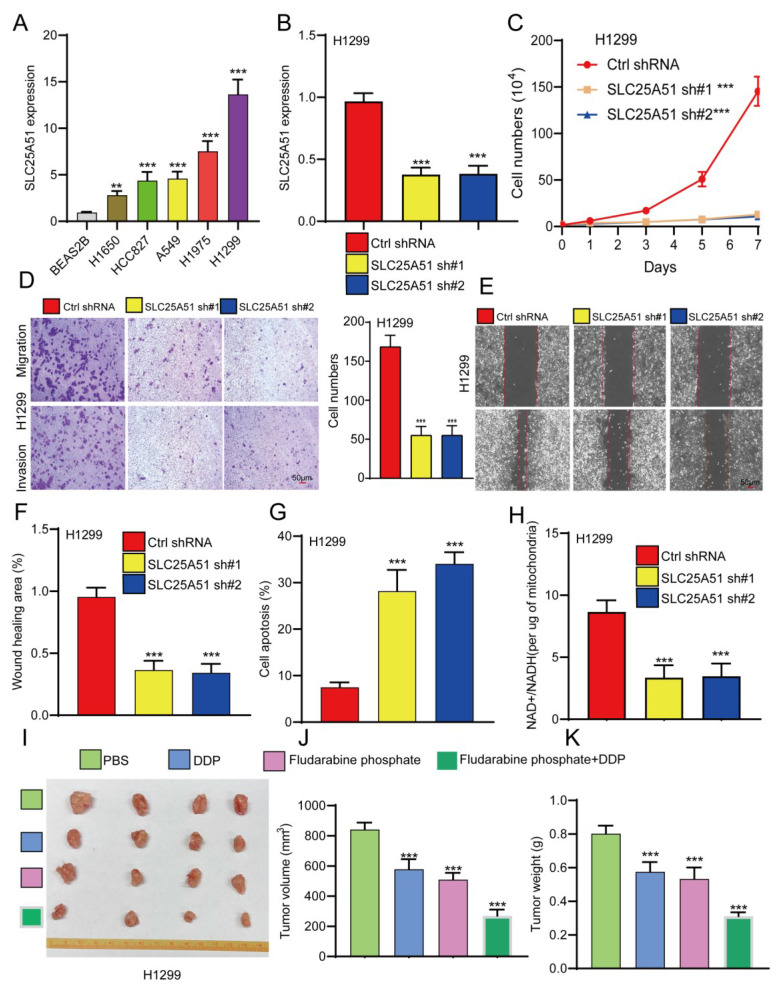
** Knockdown of SLC25A51 suppresses NSCLC progression.** (A) qPCR analysis of SLC25A51 expression in lung cancer cell lines compared to normal lung epithelial cells. (B) qPCR analysis confirming SLC25A51 knockdown efficiency in lung cancer cell lines. (C) CCK-8 assays assessing the effect of SLC25A51 knockdown on cell proliferation. (D-F) Transwell and wound healing assay assessing the effects of SLC25A51 knockdown on migration and invasion. (G) Flow cytometry analysis of apoptosis in lung cancer cells following SLC25A51 knockdown. (H) Changes in MitoSOX fluorescence intensity and NAD⁺/NADH ratio in SLC25A51 knockdown cell lines. (I-K) fludarabine phosphate effectively inhibited NSCLC tumor growth, combined treatment with fludarabine phosphate and cisplatin (DDP) resulted in a more pronounced suppression of tumor growth, as evidenced by reduced tumor volume and size, Representative images of xenograft tumors (J), tumor volume measurements (F), and tumor weights (K) are shown. (n = 4 mice per group) Data are presented as mean ± SD (bar plots). *P < 0.05; **P < 0.01; ***P < 0.001; ns not significant.

Given the lack of prior studies reporting the role and mechanism of SLC25A51 in NSCLC, we further investigated its functional significance in NSCLC progression. qPCR analysis revealed that SLC25A51 expression was significantly upregulated in the majority of NSCLC cell lines compared to normal lung epithelial cells, with the highest expression observed in H1299 cells **(Figure 5A)**. To evaluate its biological function, we established stable SLC25A51 knockdown cell lines using lentiviral shRNA constructs. qPCR confirmed efficient knockdown of SLC25A51 by two independent shRNAs **(Figure 5B)**. Functional assays demonstrated that silencing SLC25A51 markedly inhibited NSCLC cell proliferation **(Figure 5C)**. Interestingly, knockdown of SLC25A51 also significantly suppressed cell migration and invasion, and induced apoptosis in NSCLC cells **(Figure 5D-5G)**. Given that SLC25A51 functions as a mitochondrial NAD⁺ transporter, we further examined its impact on mitochondrial metabolism and observed a significant reduction in the mitochondrial NAD⁺/NADH ratio upon SLC25A51 depletion. Previous studies have reported that fludarabine phosphate can target SLC25A51 and suppress the mitochondrial NAD⁺/NADH ratio **(Figure 5H)**. We thus investigated whether fludarabine phosphate could reverse cisplatin resistance in NSCLC cells. Xenograft experiments revealed that fludarabine phosphate effectively inhibited NSCLC tumor growth. Moreover, combined treatment with fludarabine phosphate and cisplatin (DDP) resulted in a more pronounced suppression of tumor growth, as evidenced by reduced tumor volume and size **(Figure 5I-5K)**. Taken together, these findings indicate that SLC25A51 promotes NSCLC tumor progression, and that targeting SLC25A51 may represent a promising therapeutic strategy for the treatment of lung cancer.

### LINC02802 regulates mitochondrial oxidative phosphorylation

**Figure 6:**
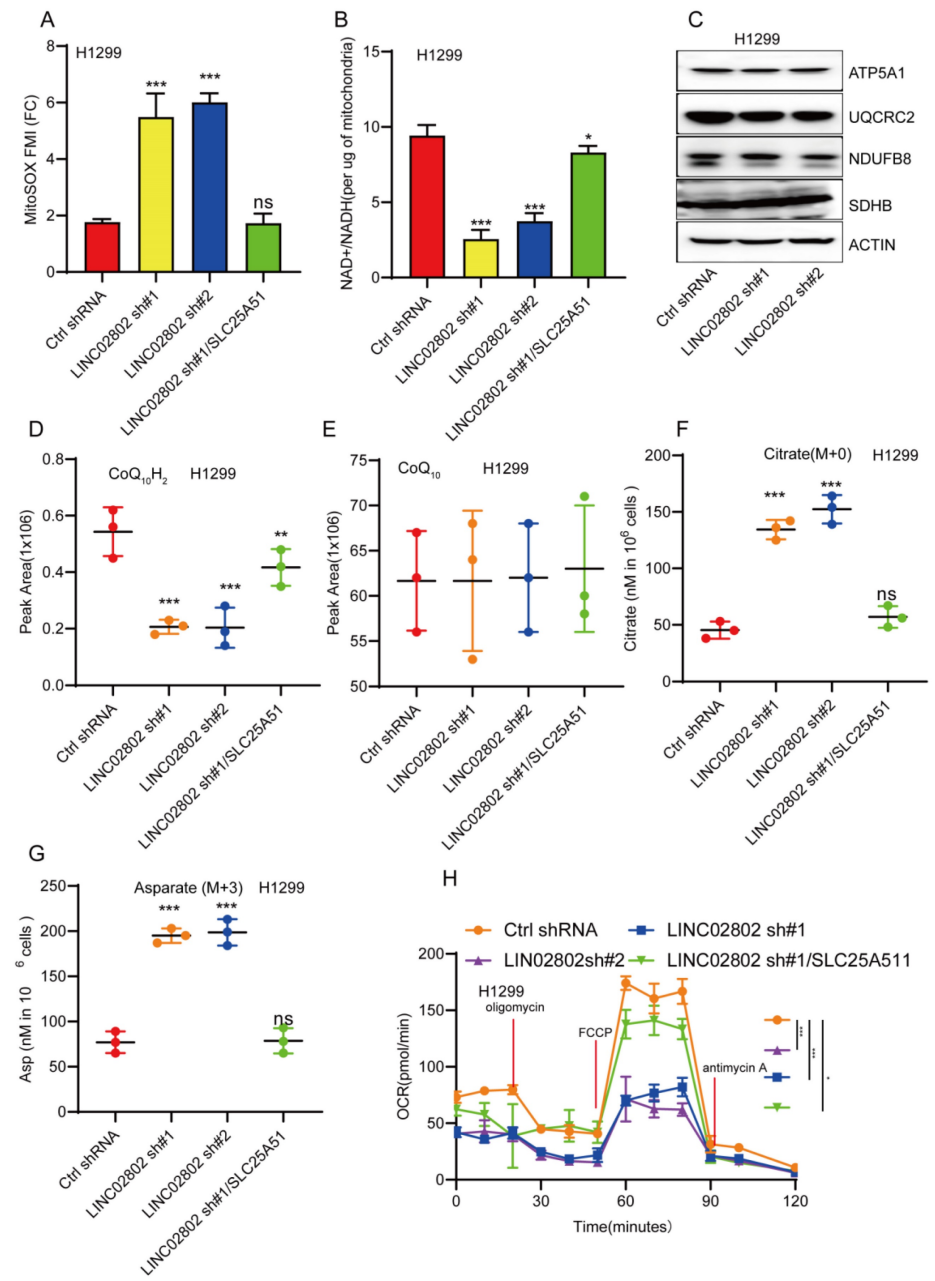
** LINC02802 regulates mitochondrial metabolism by modulating SLC25A51 expression.** (A-B) Changes in MitoSOX fluorescence intensity and NAD⁺/NADH ratio in LINC02802 knockdown cell lines. (C) Western blot analysis of mitochondrial electron transport chain complex proteins after LINC02802 knockdown. (D-G) Alterations in tricarboxylic acid (TCA) cycle intermediates in LINC02802 knockdown cell lines. (H) Assessment of mitochondrial respiratory capacity in LINC02802 knockdown cell lines. Data are presented as mean ± SD (bar plots). sh#1=LINC02802 shRNA#1, sh#2=LINC02802 shRNA#2, Ove=LINC02802 Overexpression. NC= negative control,*P < 0.05; **P < 0.01; ***P < 0.001; ns not significant.

SLC25A51 has been identified as the principal mitochondrial NAD⁺ transporter in mammalian cells, playing a critical role in maintaining the mitochondrial NAD⁺ pool. This pool is essential for various metabolic processes, including oxidative phosphorylation, the tricarboxylic acid (TCA) cycle, and mitochondrial protein deacetylation. Given that SLC25A51 is a downstream target gene of LINC02802, we further investigated whether LINC02802 regulates the mitochondrial NAD⁺ transporter. Our experiments revealed that knockdown of LINC02802 led to elevated mitochondrial superoxide levels **(Figure 6A)**, suggesting that alterations in the redox state may impair or reverse the flux of mitochondrial dehydrogenases and electron transport chain (ETC) complexes. This hypothesis was supported by the observation that LINC02802 depletion reduced the mitochondrial NAD⁺/NADH ratio and caused a specific loss of reduced ubiquinol in H1299 cells, without affecting the protein levels of ETC complexes **(Figures 6B-6E)**. These findings indicate that depletion of LINC02802 impairs oxidative capacity and disrupts electron flow through the ETC. As NSCLC cells are dependent on glutaminolysis, we performed isotope tracing using [U-¹³C, U-¹⁵N]-glutamine in proliferating H1299 cells. After 20 hours of metabolic equilibrium, we compared the incorporation of labeled metabolites between LINC02802-depleted and control cells. We observed a significant increase in the use of non-glutamine carbon sources to support the TCA cycle in LINC02802-knockdown cells, as evidenced by a higher proportion of unlabeled TCA intermediates **(Figure 6F and Figures S5A-S5C).** Furthermore, LINC02802 was found to be essential for maintaining oxidative TCA cycle flux **(Figures S5D-S5G),** and its depletion led to increased levels of M+5-labeled citrate, indicating enhanced reductive flux. This metabolic shift was particularly evident in M+3-labeled aspartate **(Figure 6G and Figure S5H)**. Additionally, knockdown of LINC02802 reduced mitochondrial respiratory capacity, an effect that was rescued by overexpression of SLC25A51** (Figure 6H)**. Collectively, these results demonstrate that LINC02802 regulates the oxidative state of the mitochondrial NAD⁺/NADH ratio by modulating SLC25A51 expression, thereby controlling TCA cycle metabolic flux. Thus, the LINC02802/SLC25A51 axis plays a crucial role in regulating oxidative TCA metabolism in NSCLC cells.

### LINC02802 promotes NSCLC progression by regulating SLC25A51 expression

**Figure 7:**
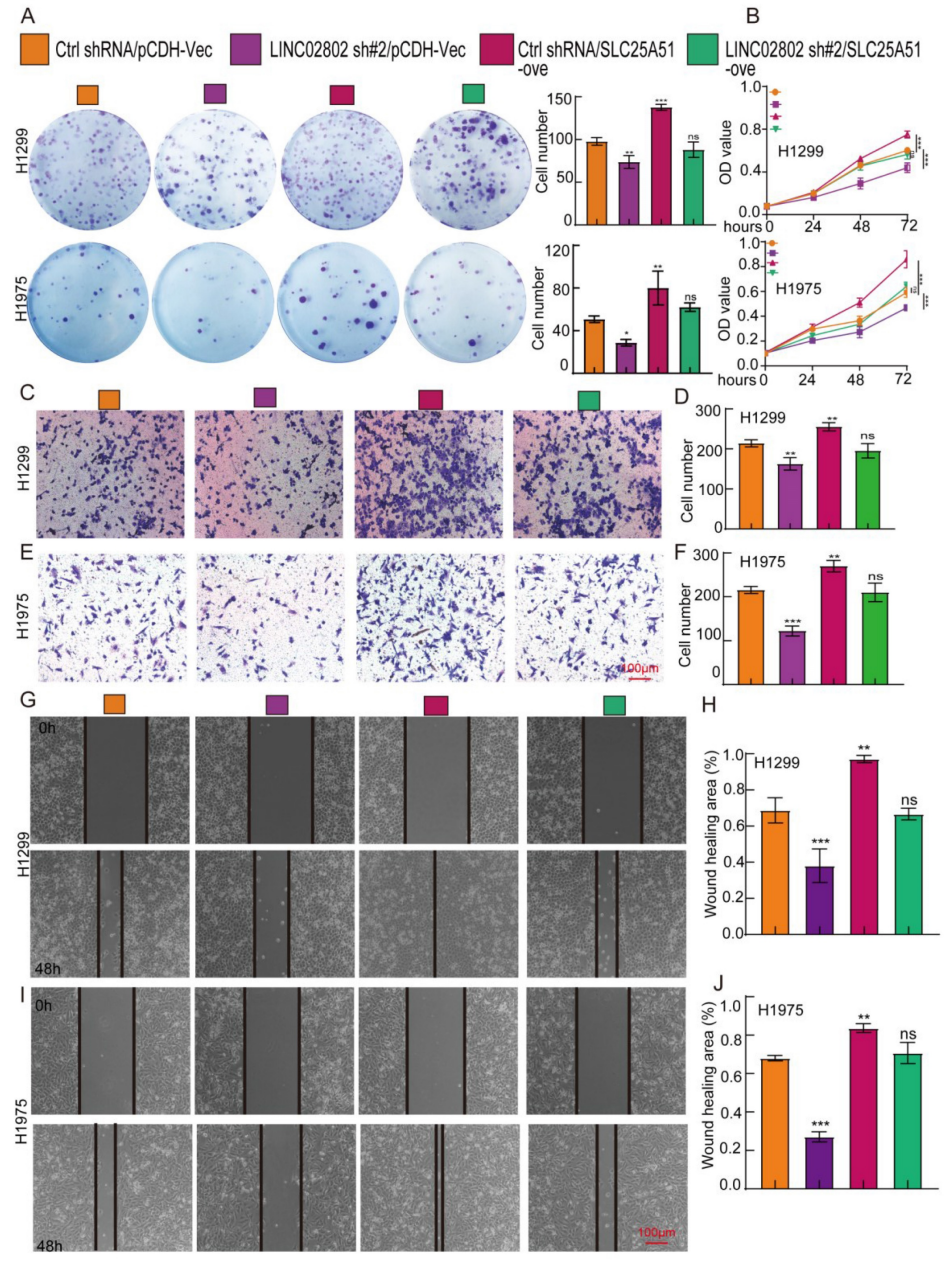
** LINC02802 promotes malignant phenotypes of lung cancer cells by upregulating SLC25A51 expression.** (A-B) Clonogenic and CCK-8 assays demonstrating the role of the LINC02802/SLC25A51 axis in promoting lung cancer cell proliferation. (C-D) Transwell assays showing that the LINC02802/SLC25A51 axis enhances the migratory capacity of lung cancer cells. (G-J) Wound healing assays further validating the involvement of the LINC02802/SLC25A51 axis in lung cancer cell migration. sh#1=LINC02802 shRNA#1, sh#2=LINC02802 shRNA#2, Ove=LINC02802 Overexpression. Data are presented as mean ± SD (bar plots). *P < 0.05; **P < 0.01; ***P < 0.001; ns not significant.

To further confirm that SLC25A51 is a downstream target gene of LINC02802 in promoting lung cancer progression, we performed cell biology rescue experiments. The rescue experiments showed that knockdown of LINC02802 significantly inhibited the proliferative capacity of lung cancer cells, as validated by CCK8 assays and colony formation assays, yielding consistent results across two lung cancer cell lines. Moreover, overexpression of SLC25A51 was able to rescue the inhibitory effects of LINC02802 knockdown on the proliferation and growth capabilities of lung cancer cells** (Figure 7A-7B).** Knockdown of LINC02802 also suppressed the migration and invasion abilities of lung cancer cells, and these results were confirmed through transwell and scratch assays. Importantly, overexpression of SLC25A51 could reverse the inhibitory effects of LINC02802 knockdown on the migration and invasion capacities of lung cancer cells** (Figure 7C-7J)**. In summary, this evidence partially indicates that LINC02802 functions to promote tumor progression by regulating the expression of SLC25A51.

### Targeting LINC02802 reverses cisplatin resistance in NSCLC cells

**Figure 8:**
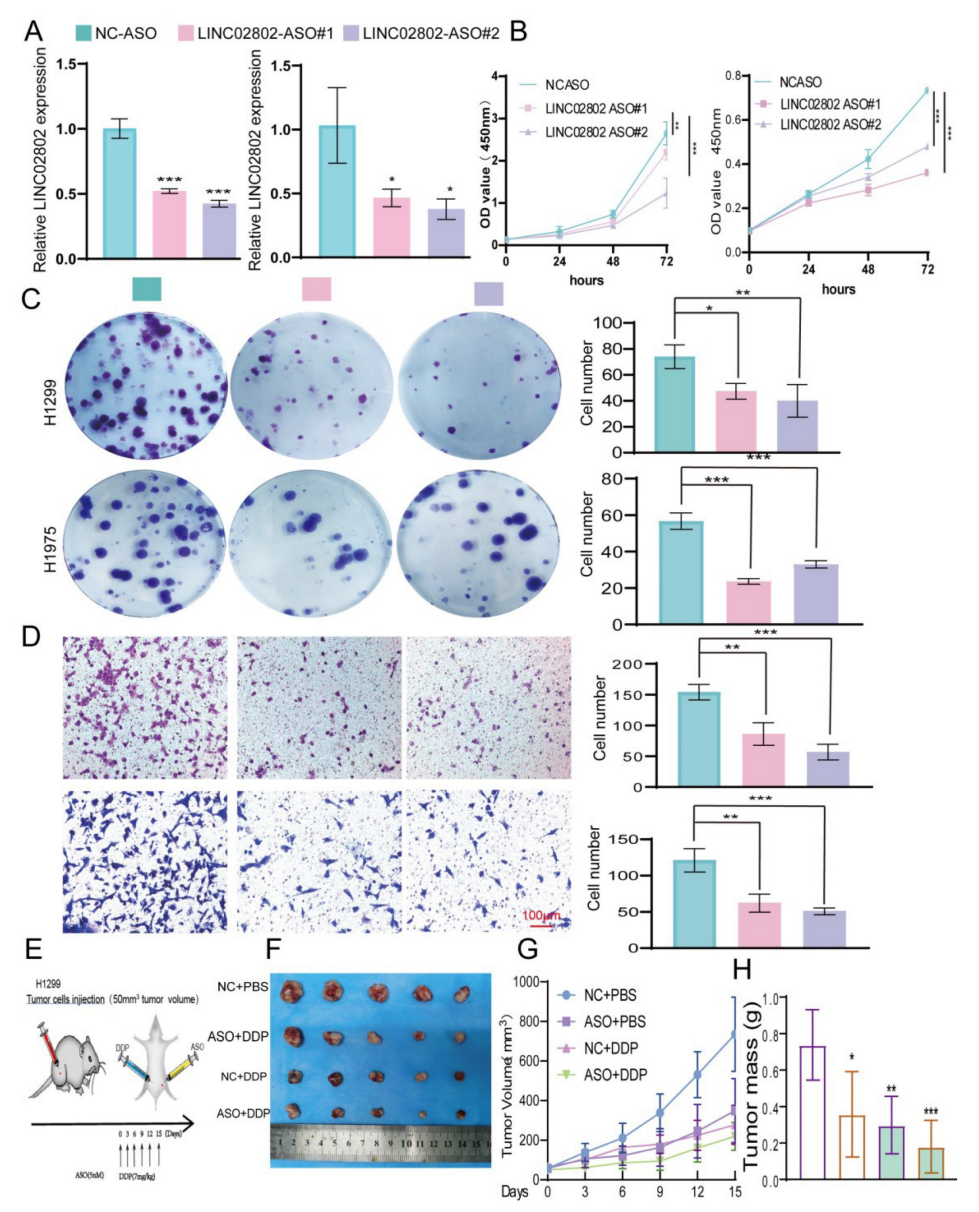
** Targeting LINC02802 reverses cisplatin resistance in lung cancer cells.** (A) qPCR analysis confirming the knockdown efficiency of LINC02802-specific antisense oligonucleotides (ASOs) in lung cancer cells. (B) CCK-8 assay showing the inhibitory effect of LINC02802-specific ASOs on cell proliferation. (C) Colony formation assay indicating reduced colony-forming ability following ASO treatment. (D) Transwell assay demonstrating decreased migration and invasion of lung cancer cells upon LINC02802 knockdown. (E-G) LINC02802 knockdown suppresses tumor growth *in vivo*. Representative images of xenograft tumors (E), tumor volume measurements (F), and tumor weights (G) are shown. (n = 5 mice per group) Data are presented as mean ± SD (bar plots). *P < 0.05; **P < 0.01; ***P < 0.001; ns not significant.

Studies have shown that Antisense Oligonucleotides (ASOs) inhibit specific gene expression by binding complementarily to specific mRNA sequences, preventing mRNA translation. ASOs have broad application prospects in treating genetic diseases, cancer, and viral infections. To further explore the impact of targeting LINC02802 on lung cancer cell growth, we designed specific ASOs targeting LINC02802 and validated their function both *in vitro*. *In vitro* functional experiments demonstrated that LINC02802-specific ASOs significantly inhibited LINC02802 expression** (Figure 8A-8D).**

To evaluate the therapeutic potential of LINC02802 silencing *in vivo*, we conducted a xenograft experiment using H1299 cells and treated tumor-bearing mice with antisense oligonucleotides targeting LINC02802 and cisplatin, either alone or in combination **(Figure 8E).** The combination of ASO and DDP resulted in the most pronounced suppression of tumor growth, as evidenced by smaller and fewer tumors **(Figure 8F),** significantly reduced tumor volumes **(Figure 8G)**, and lower tumor weights **(Figure 8H)**, compared to other treatment groups. These results demonstrate that targeting LINC02802 enhances the anti-tumor efficacy of cisplatin in lung cancer, suggesting that LINC02802 inhibition may be a promising strategy to improve chemotherapeutic outcomes.

### NAT10 upregulates LINC02802 expression through Ac4C modification

We further explored the mechanism underlying the aberrant upregulation of LINC02802 in lung adenocarcinoma. Numerous studies have demonstrated that RNA epigenetic modifications, including m6A, m5C, and Ac4C, play crucial roles in regulating RNA stability 21. Initially, through database analysis, we found that LINC02802 lacks significant m6A modification sites. Moreover, analysis of our previously published m6A-RIP-seq data from lung cancer cell lines revealed no enrichment of LINC02802 by the m6A antibody, suggesting that the expression of LINC02802 may not be associated with m6A modification (data not shown). We also analyzed previously published RNA m5C-seq data, and the results showed that there is no significant m5C modification on the LINC02802 transcript (data not shown).

**Figure 9:**
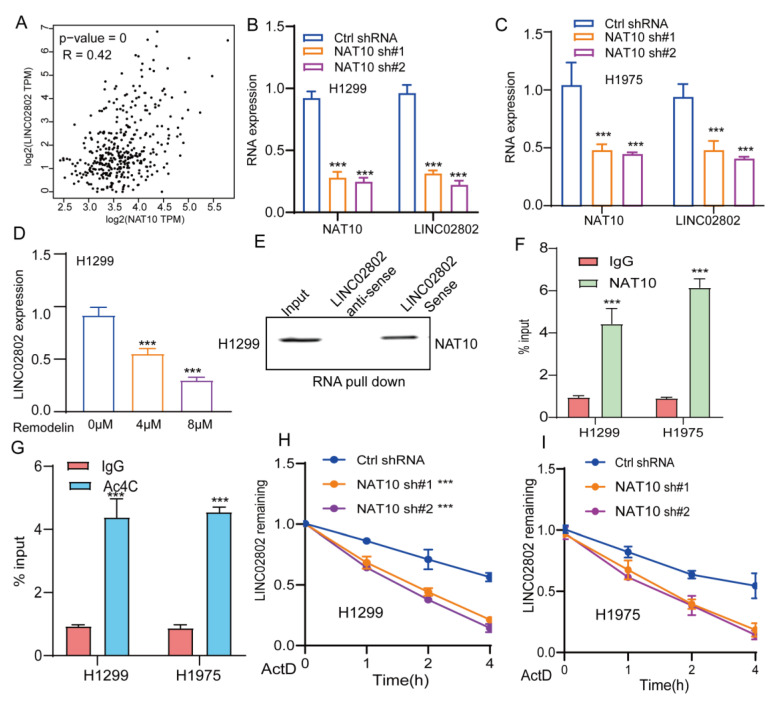
** NAT10 mediates Ac4C modification of LINC02802, contributing to its stability.** (A) TCGA-based correlation analysis of NAT10 and LINC02802 expression in LUAD tissues. (B-C) qPCR analysis showing decreased LINC02802 expression following NAT10 knockdown. (D) qPCR results of LINC02802 levels after treatment with a NAT10 inhibitor. (E-F) RNA pull-down and RIP-qPCR assays validating the interaction between NAT10 and LINC02802. (G) Ac4C-RIP-qPCR assays confirming the presence of Ac4C modification on LINC02802 transcripts. (H-I) RNA stability assays showing a reduced half-life of LINC02802 following NAT10 knockdown. Data are presented as mean ± SD (bar plots). *P < 0.05; **P < 0.01; ***P < 0.001; ns not significant.

Ac4C (N4-acetylcytidine) is an RNA modification found on both mRNAs and lncRNAs. In recent years, accumulating research has highlighted its pivotal role in regulating RNA stability. Ac4C modification is primarily catalyzed by N-acetyltransferase 10, which plays a critical role in regulating the stability, translation efficiency, and function of lncRNAs^22^, ^23^. Recent studies have shown that NAT10-mediated Ac4C modification enhances the stability of lncRNA SIMALR and lncRNA CTC-490G23.2, promoting malignant progression in nasopharyngeal carcinoma and esophageal squamous cell carcinoma, respectively 24, 25. Previous research has also revealed that NAT10 is significantly upregulated in non-small cell lung cancer, where it promotes tumor progression 26. Our analysis further revealed a highly significant positive correlation between the expression of NAT10 and LINC02802 suggesting that NAT10 may play a key role in regulating LINC02802 expression **(Figure 9A)**. We found that knockdown of NAT10 significantly reduced the expression of LINC02802 **(Figure 9B-9C)**, and treatment of lung cancer cells with the NAT10 inhibitor remodelin also downregulated LINC02802 expression **(Figure 9D)**. Further RNA pull-down and RIP experiments demonstrated that LINC02802 and NAT10 could bind to each other **(Figure 9E-9F)**, and Ac4C-RIP experiments confirmed the presence of significant Ac4C modification on LINC02802 **(Figure 9G)**. RNA stability assays showed that knockdown of NAT10 significantly reduced the RNA stability of LINC02802 **(Figure 9H-9I)**. In summary, these findings suggest that NAT10 mediates Ac4C modification of LINC02802, enhancing its stability and contributing to its aberrant upregulation in LUAD.

## Discussion

Non-small cell lung cancer remains a leading cause of cancer-related mortality worldwide, with limited effective treatment options, particularly in the context of metabolic reprogramming 27. Our study identifies LINC02802 as a novel oncogenic lncRNA that promotes NSCLC progression through metabolic modulation. Mechanistically, LINC02802 functions as a molecular sponge for miR-1976, a microRNA that targets and suppresses SLC25A51. The derepression of SLC25A51 enhances the import of NAD+ into mitochondria, elevating the NAD+/NADH ratio and promoting oxidative metabolism through the TCA cycle. This metabolic shift supports the energetic and biosynthetic demands of rapidly proliferating cancer cells.

Importantly, our results extend the biological relevance of lncRNA-mediated ceRNA networks into the realm of mitochondrial metabolism, an area of growing interest in tumor biology. The rescue of impaired proliferation and mitochondrial respiration by SLC25A51 re-expression in LINC02802-silenced cells highlights the centrality of this axis. Furthermore, our use of MitoLbNox to mimic oxidative NAD+/NADH conditions underscores the critical role of redox balance in NSCLC progression. These findings align with recent insights into the significance of mitochondrial NAD+ dynamics in cancer and suggest that modulating this balance may represent a viable strategy for therapeutic intervention. SLC25A51 has been identified as the primary transporter responsible for the selective import of NAD⁺ into mitochondria, playing a central role in maintaining mitochondrial NAD⁺/NADH homeostasis. Loss of SLC25A51 significantly disrupts mitochondrial NAD⁺ levels, impairing oxidative phosphorylation and cellular metabolism. Although SLC25A52, a close paralog of SLC25A51, has been proposed as a potential compensatory transporter, its expression is typically much lower and insufficient to fully restore mitochondrial NAD⁺ transport in most cell types. Thus, SLC25A51 appears to be functionally non-redundant under physiological conditions, and its activity is critical for mitochondrial redox balance and metabolic function. While our metabolic analysis primarily relies on [U-¹³C]-glutamine tracing to assess the role of LINC02802 and SLC25A51 in NSCLC metabolism, we acknowledge that incorporating additional tracers such as [U-¹³C]-glucose or [U-¹³C]-acetate would provide a more comprehensive view of carbon flux and help rule out alternative metabolic pathways. Future studies employing multiple isotopic tracers will be essential to fully delineate the metabolic rewiring induced by LINC02802 in lung cancer. Nonetheless, our current data offer strong evidence that glutamine metabolism plays a critical role in LINC02802-mediated metabolic regulation.

Antisense oligonucleotides (ASOs) targeting long non-coding RNAs (lncRNAs) have made significant progress in cancer research, emerging as a novel strategy for cancer treatment 28. ASOs are short nucleic acid molecules that can specifically bind to target RNA, influencing the biological behavior of cells by modulating target RNA expression or function 29. Our previous studies found that ASOs targeting PKMYT1AR could significantly inhibit the self-renewal capability of lung cancer stem cells and suppress lung tumor growth 30. This study further demonstrates that LINC02802 plays a pivotal role in mediating cisplatin resistance in non-small cell lung cancer. Targeted inhibition of LINC02802 using antisense oligonucleotides significantly restored cisplatin sensitivity in resistant NSCLC cells, suggesting that LINC02802 is a key regulatory factor in the development of drug resistance.

Mechanistically, LINC02802 functions as a competing endogenous RNA by sponging miR-1976, thereby relieving the suppression of its downstream target SLC25A51. This axis maintains mitochondrial NAD⁺/NADH homeostasis and supports oxidative phosphorylation. ASO-mediated silencing of LINC02802 disrupts this metabolic balance, sensitizing tumor cells to cisplatin and offering a promising therapeutic strategy to overcome chemoresistance in NSCLC. Although our ROC curve analysis suggests that LINC02802 may serve as a promising diagnostic biomarker for NSCLC, the relatively limited cohort size and lack of external validation warrant caution in interpreting these findings. Additionally, while the anti-sense oligonucleotide (ASO) therapy showed efficacy in peri-tumoral delivery, the systemic therapeutic potential and safety profile remain to be fully evaluated. Future studies with larger patient cohorts and systemic delivery models are needed to substantiate the diagnostic and therapeutic relevance of LINC02802 in clinical settings. Fludarabine phosphate, an FDA-approved drug, has been shown to bind to and inhibit SLC25A51, leading to a decrease in mitochondrial NAD⁺ levels and subsequent protein hyperacetylation 15.

NAT10 is a key enzyme responsible for catalyzing the N4-acetylcytidine (ac4C) modification in RNA. This ac4C modification, primarily observed in mRNA, tRNA, and lncRNA, plays a significant role in regulating RNA stability and function 31. In our study, we found that NAT10-mediated Ac4C modification, rather than m6A modification, plays a crucial role in maintaining the stability of LINC02802, which partially explains the upregulation of LINC02802 in NSCLC. Given that LINC02802 regulates the mitochondrial NAD⁺/NADH ratio, and mitochondrial metabolism is closely intertwined with immune cell activation and function, it is plausible that the tumor immune microenvironment (TIME) may contribute to the upregulation of LINC02802 in lung cancer. Specifically, immune-suppressive components of the TIME—such as regulatory T cells (Tregs), tumor-associated macrophages (TAMs), and elevated levels of cytokines like TGF-β and IL-10—may induce metabolic reprogramming that favors LINC02802 expression. Furthermore, hypoxia and nutrient competition within the TIME may further enhance LINC02802-mediated adaptation of cancer cell metabolism. Future studies should explore whether specific immune cell subsets or cytokine signaling pathways directly influence LINC02802 transcriptional regulation in the lung tumor microenvironment.

Our study highlights the critical role of SLC25A51 as a mitochondrial NAD⁺ transporter that governs key aspects of cellular metabolism in lung cancer. By regulating the mitochondrial NAD⁺/NADH ratio, SLC25A51 directly influences oxidative phosphorylation and energy production, processes that are often reprogrammed during tumor progression. Dysregulation of mitochondrial metabolism through altered SLC25A51 activity can enhance cancer cell proliferation, survival, and metastatic potential. Importantly, the modulation of mitochondrial NAD⁺ homeostasis by SLC25A51 also appears to contribute to therapy resistance, particularly to platinum-based chemotherapy such as cisplatin. Cancer cells with elevated SLC25A51 expression may sustain mitochondrial function and redox balance, thereby mitigating chemotherapy-induced oxidative stress and apoptosis. Conversely, targeting SLC25A51 disrupts this metabolic resilience, sensitizing tumor cells to treatment. Thus, SLC25A51 represents not only a metabolic regulator fundamental to cancer cell fitness but also a promising target to overcome therapy resistance. Future therapeutic strategies that inhibit SLC25A51 function or its downstream metabolic pathways could enhance the efficacy of existing cancer treatments and improve patient outcomes.

## Conclusion

In summary, our study reveals a novel regulatory axis in NSCLC involving NAT10-mediated stabilization of LINC02802, which acts as a competing endogenous RNA (ceRNA) to sponge miR-1976, thereby de-repressing its downstream target SLC25A51. Upregulation of SLC25A51 enhances mitochondrial NAD⁺/NADH balance and oxidative phosphorylation, ultimately promoting cell proliferation and migration while inhibiting apoptosis, contributing to NSCLC progression. These findings highlight the critical role of the LINC02802/miR-1976/SLC25A51 axis in mitochondrial metabolism and lung cancer development, providing new insights into potential therapeutic targets for NSCLC.

## Supplementary Material

Supplementary figures and tables.

## Abbreviations

NSCLC: Non-small cell lung cancer
SLC25A51: Solute Carrier Family 25 Member 51
LUAD: Lung adenocarcinoma
LUSC: Lung squamous cell carcinoma
ceRNA: Competing endogenous RNA
LncRNA: Long non-coding RNA
WT: Wild-type
DDP: Cisplatinum
ROC: Receiver operating characteristic
AUC: Area under the curve
ASO: Antisense oligonucleotide
3'-UTR: 3'-untranslated region
OS: Overall survival
qRT-PCR: Quantitative reverse transcription polymerase chain reaction
FISH: Fluorescence *in situ* hybridization
EMT: Epithelial-mesenchymal transition
miRNA: MicroRNA
shRNA: Short hairpin RNAs
FBS: Fetal calf serum
GEO: Gene Expression Omnibus
TCGA: The Cancer Genome Atlas
PBS: Phosphate-buffered saline
OXPHOS: Oxidative phosphorylation
