# Supplementary material for: N4-acetylcytidine modification of LINC02802 promotes non-small cell lung cancer progression by modulating mitochondrial NAD+/NADH ratio
Source: Int J Biol Sci. 2025 Jul 28;21(11):4908–26. doi: 10.7150/ijbs.116639 (PMC12374817; doi:10.7150/ijbs.116639)
Supplement: Supplementary file 1 — Supplementary figures and tables. [file ijbsv21p4908s1.pdf]

Supplementary Materials for

N4-acetylcytidine modification of LINC02802 promotes non-small cell lung cancer progression by modulating mitochondrial NAD+/NADH ratio

Yixiao Yuan[1,2#,] Dahang Zhang[1#], Juan Wang[1#], Lin Tang[1#], Yaowu Duan[1], Lincan Duan[3*], Xiulin Jiang[2*],

1、Department of Thoracic Surgery, Yunnan Cancer Hospital, The Third Affiliated Hospital of Kunming Medical University, Peking University Cancer Hospital Yunnan, Kunming, 650118, China.

2、Department of Systems Biology, Beckman Research Institute of City of Hope, Monrovia, CA 91016, USA;

3、Department of Thoracic Surgery, Pu'er People's Hospital, Pu'er, China.

[#]Contributed equally

[*]Corresponding author:

Xiulin Jiang:xiulinjiang17@163.com

Lincan Duan:duanmumuhuosan@163.com

Running title: Role of LINC02802 in NSCLC

This PDF file includes:

Materials and Methods

1、Figures. S1-S4

2、Table S1-S3

Supplementary Figure and Figure legend

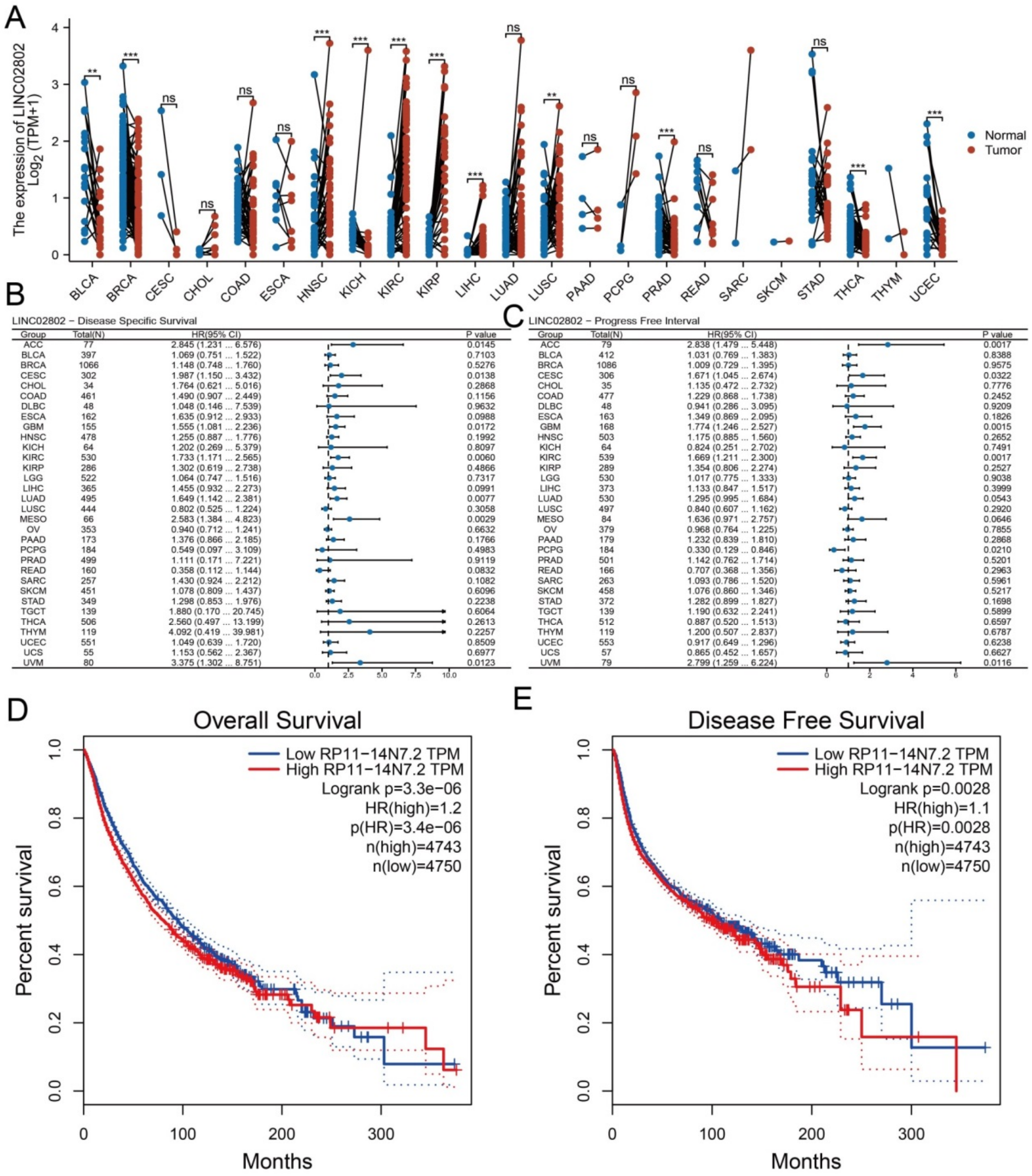

Figure S1 The expression and prognosis of LINC02802 in pan-cancer.

(A) Integration of TCGA and GTEx databases to analyze the expression of LINC02802 across various cancers. (B-E) Validation of the prognostic significance of LINC02802 across various cancers using the TCGA dataset. *P < 0.05, **P < 0.01 and ***P < 0.001.

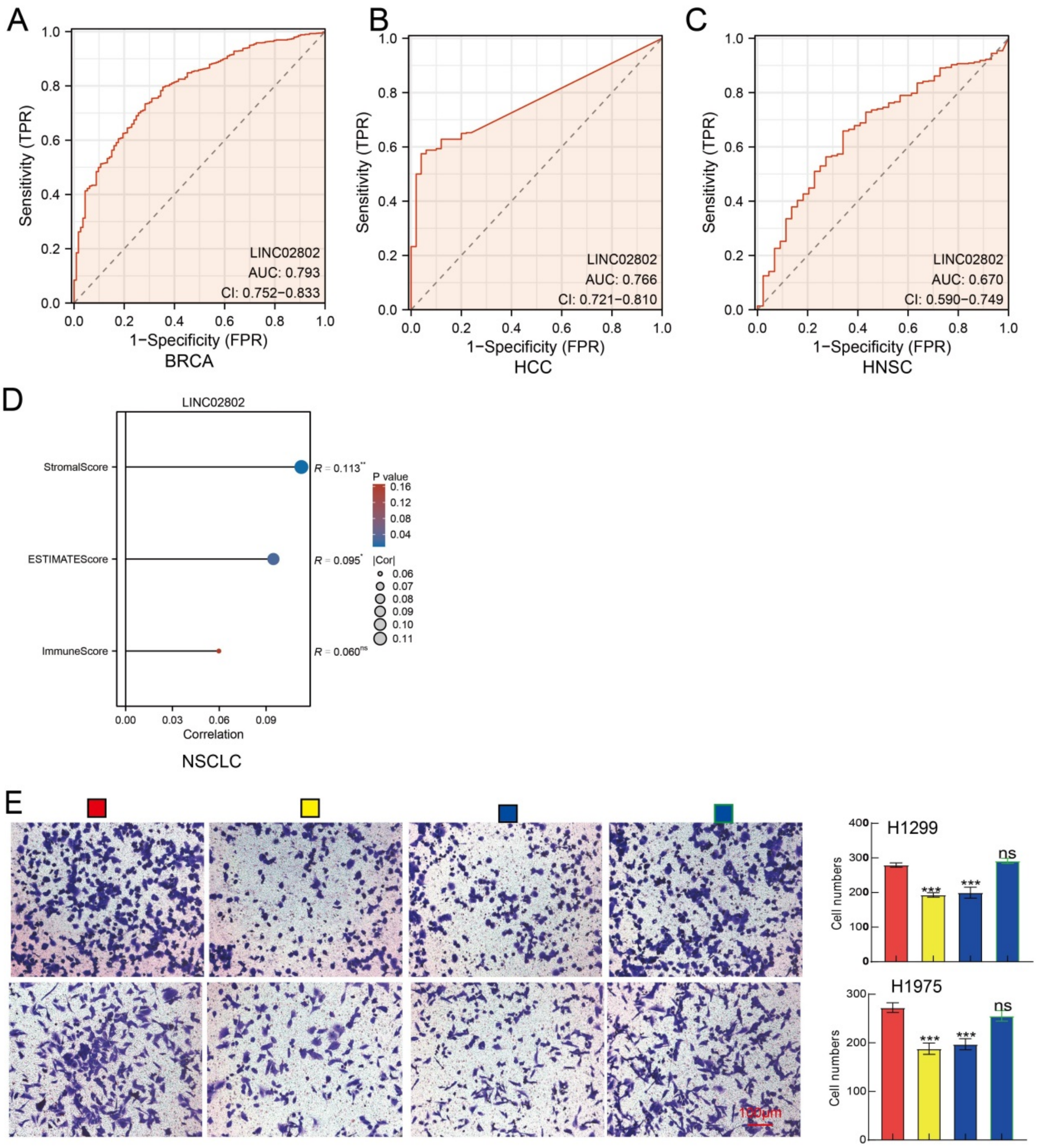

Figure S2 The ROC curve of LINC02802 in other cancers was analyzed using the TCGA dataset. (A-C) ROC curve analysis evaluating the diagnostic performance of LINC02802 in HCC, BRCA and HNSC. (D)Analysis of the correlation between LINC02802 expression and lung cancer tumor immune microenvironment. (E) Transwell assays assessing the effects of LINC02802 knockdown on migration and invasion. sh#1=LINC02802 shRNA#1, sh#2=LINC02802 shRNA#2, Ove=LINC02802 Overexpression. *P < 0.05, **P < 0.01, ***P < 0.001.

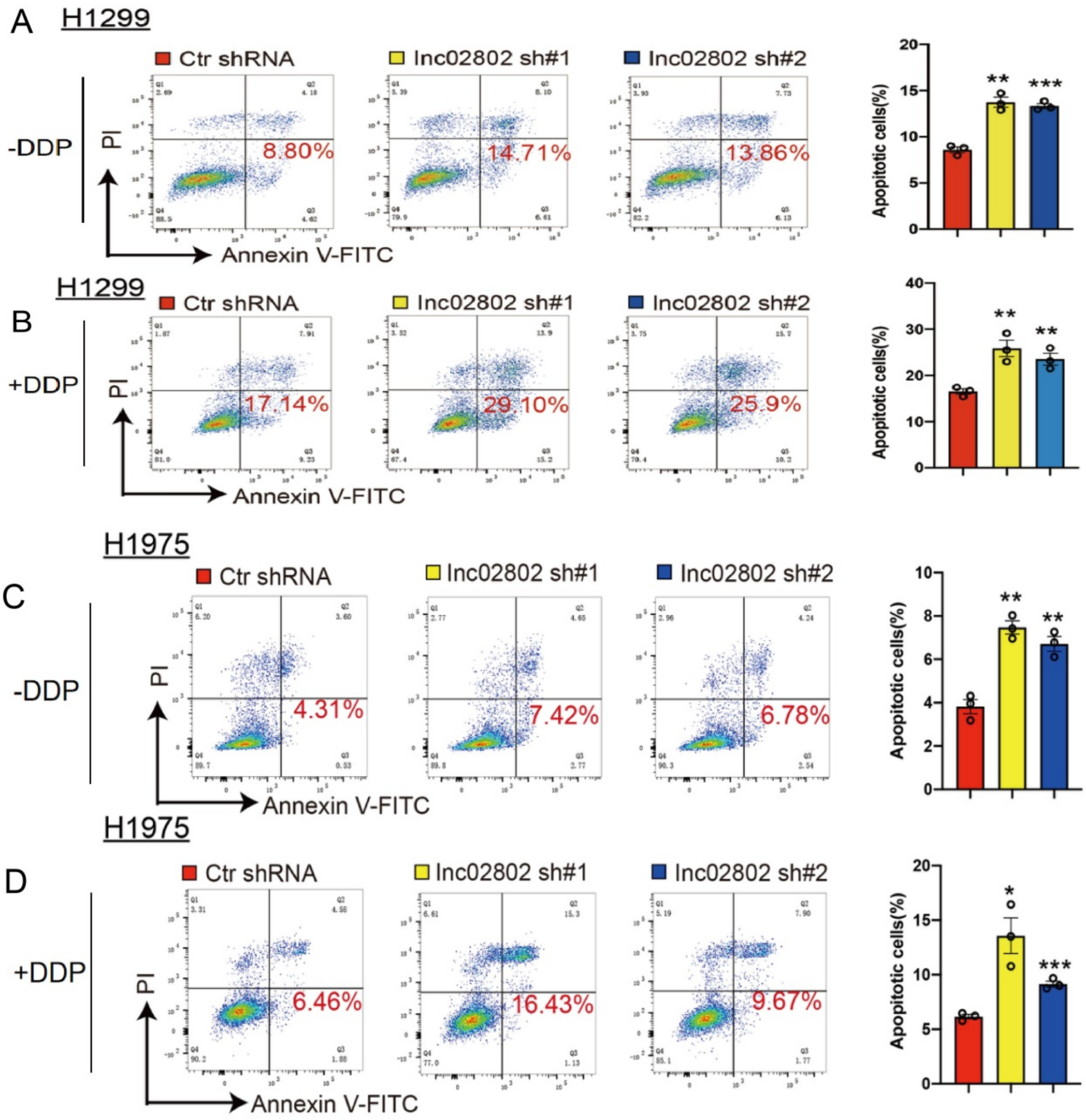

Figure S3. Knockdown of LINC02802 promotes apoptosis in lung cancer cells.

(A–D) Flow cytometry analysis of apoptosis in lung cancer cells following LINC02802 knockdown, with or without cisplatin treatment. *P < 0.05, **P < 0.01, ***P < 0.001.

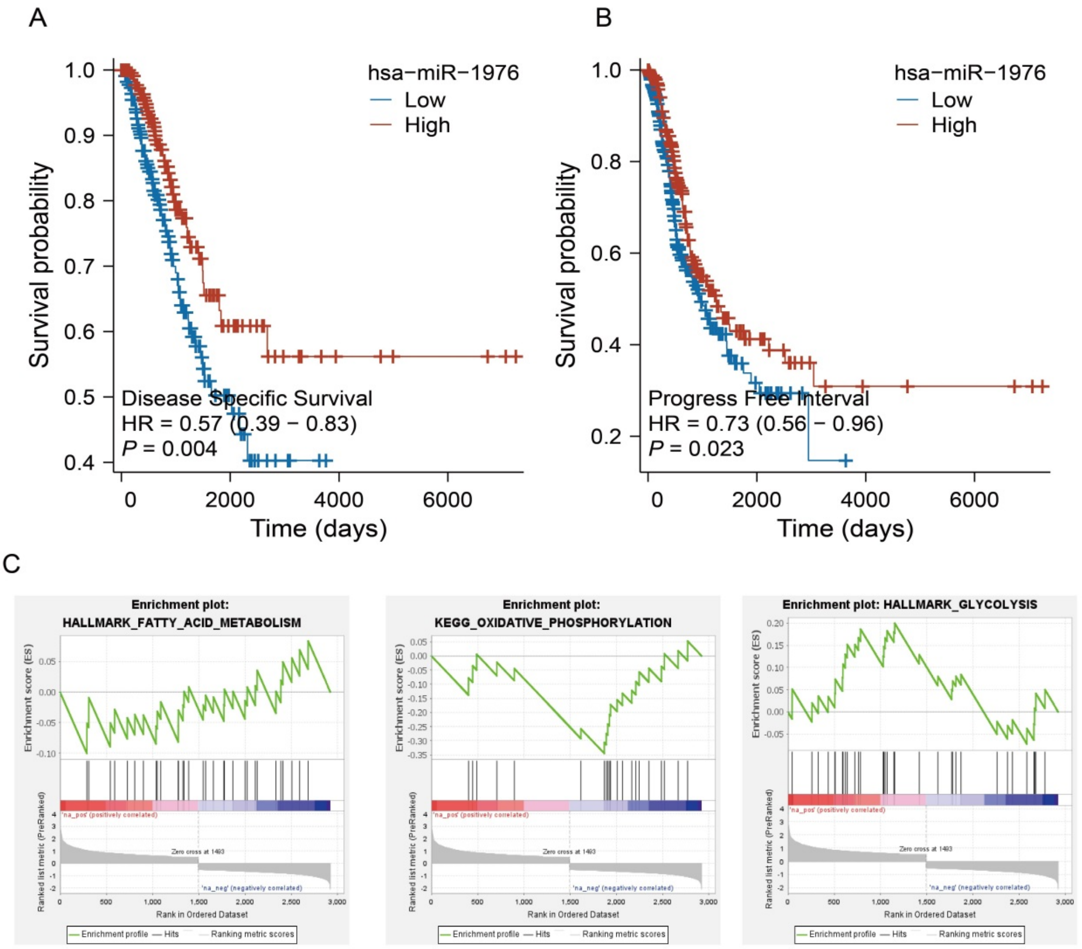

Figure S4. Pathway enrichment analysis of LINC02802-related signaling pathways.

(A–B) Prognostic analysis of miR-1976 in lung cancer. (C) Pathway enrichment analysis of differentially expressed genes based on transcriptomic profiling of LINC02802-knockdown cell lines. *P < 0.05, **P < 0.01, ***P < 0.001.

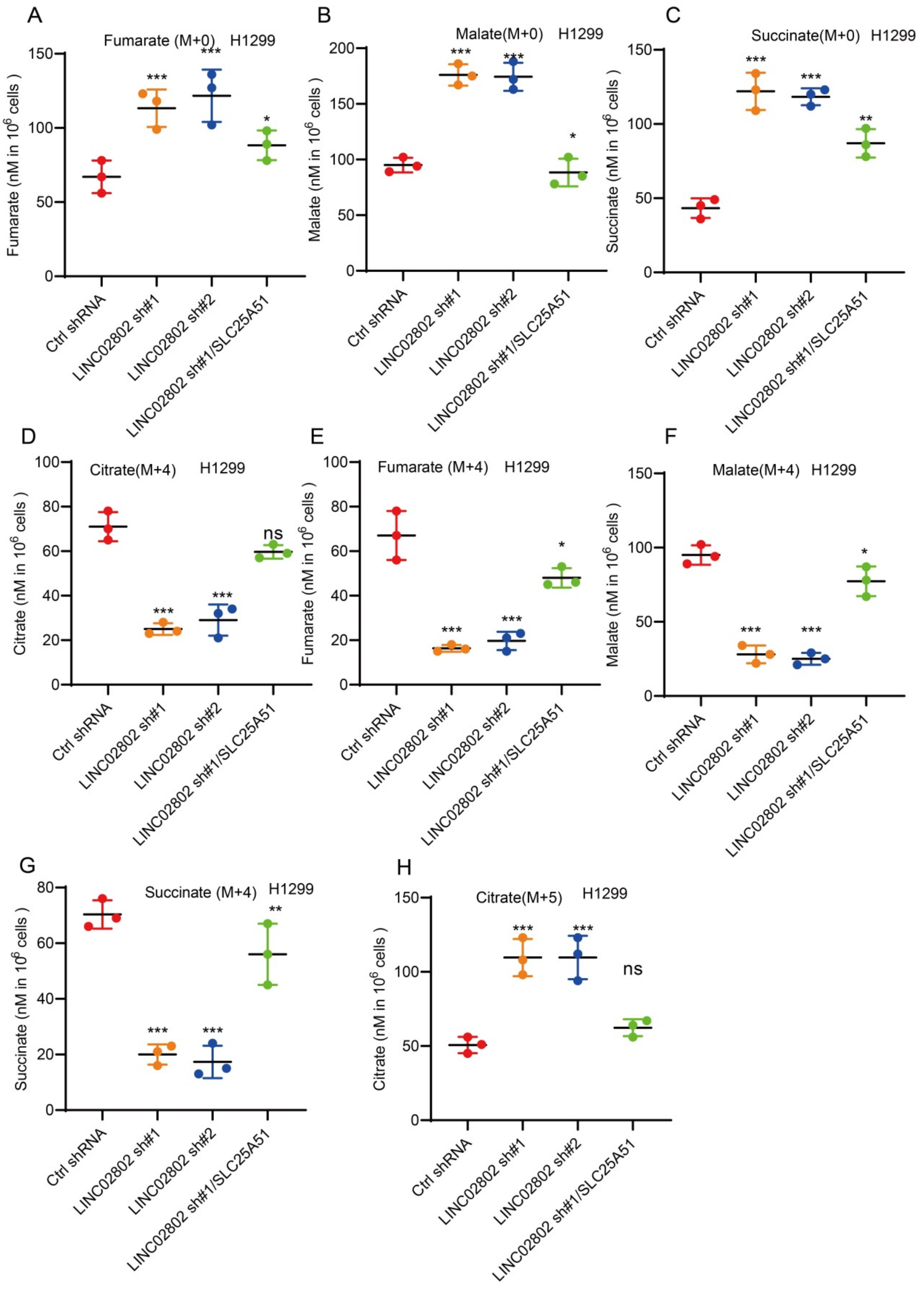

Figure S5. Assessment of TCA cycle intermediates following LINC02802 knockdown.

(A–H) Quantification of tricarboxylic acid (TCA) cycle intermediates in lung cancer cells after LINC02802 knockdown using commercially available assay kits. $*P < 0.05$, $**P < 0.01$, $***P < 0.001$.

Table S1. Primer sequences used in this study.

Primer Name	Primer Sequences(5'-3')
Human β-actin_F	AAGTGTGACGTGGACATCCGC
Human β-actin_R	CCGGACTCGTCATACTCCTGCT
LINC02802-F	CTCCCCAGCTGTGAAAGGAC
LINC02802-F	CCATGGGAAATGAAGCGACA
SLC25A51-F	GCTGCTGTGCAGCCTTCAACAATGTCGCAAT
SLC25A51-F	TCCAGTGGAGTGAAAATTGCTTCTGTTG
LINC02802-shRNA1-F	CCGGGCCTCCATAGAGCTGCCCAGCCTCGAGGCTG<br>GGCAGCTCTATGGAGGCTTTTTG
LINC02802-shRNA1-R	AATTCAAAAAGCCTCCATAGAGCTGCCCAGCCTCG<br>AGGCTGGGCAGCTCTATGGAGGC
LINC02802-shRNA2-F	CCGGGCAGACCCGCAGAGATGAATGCTCGAGCATT<br>CATCTCTGCGGGTCTGCTTTTTG
LINC02802-shRNA2-R	AATTCAAAAAGCAGACCCGCAGAGATGAATGCTCG<br>AGCATTCATCTCTGCGGGTCTGC
NAT10-F	CACGGAATATGGTGGACTATCA
NAT10-R	GTTGAAAAGTCCCATCAACTGG
NAT10-shRNA1-F	AAATAGCTTCACAACTTTGCG
NAT10-shRNA2-F	ATAGTCACTGTGTACAATTGC
LINC02802 ASO#1	CAGCATGGCTGCATCCAGTA

LINC02802 ASO#2	CAGCATGGCTGCATCCAGTA

Table S2. Antibodies used in this study.

Antibody Name	Catalog Number	Dilution	Supplier	Species
NDUFB8	14794-1-AP	1:1000	Proteintech	Rabbit
UQCRC2	14742-1-AP	1:1000	Proteintech	Rabbit
SDHB	106201-1-AP	1:1000	Proteintech	Rabbit
MTCO2	14676-1-AP	1:1000	Proteintech	Rabbit
β-actin	60008-1-1g	1:5000	Proteintech	Mouse
NAYT10	ab194297	1:1000	Abcam	Rabbit
SLC25A51	ab237054	1:1000	Abcam	Rabbit
Anti-N4-acetylcytidine (ac4C) antibody	ab252215	1:1000	Abcam	Rabbit

Table S3. Commercial assay kits used for the detection of TCA cycle intermediates.

FITC Annexin V Apoptosis Detection Kit I	BD Biosciences	# 556547
RNeasy Plus Mini Kit	Qiagen	# 74134
Mitochondria Isolation Kit, human	Miltenyi Biotec	# 130-094-532
NAD/NADH-Glo Assay	Promega	# G9071
Seahorse XFp Cell Mito Stress Test Kit	Agilent	# 103010-100
Seahorse XFp Glycolysis Stress Test Kit	Agilent	# 103017-100
